# Cerebellum Abnormalities in Idiopathic Generalized Epilepsy with Generalized Tonic-Clonic Seizures Revealed by Diffusion Tensor Imaging

**DOI:** 10.1371/journal.pone.0015219

**Published:** 2010-12-21

**Authors:** Yonghui Li, Hanjian Du, Bing Xie, Nan Wu, Jian Wang, Guocai Wu, Hua Feng, Tianzi Jiang

**Affiliations:** 1 National Research Center for Intelligent Computing Systems, Institute of Computing Technology, Chinese Academy of Sciences, Beijing, People's Republic of China; 2 National Laboratory of Pattern Recognition, LIAMA Center for Computational Medicine, Institute of Automation, Chinese Academy of Sciences, Beijing, People's Republic of China; 3 Department of Neurosurgery, Southwest Hospital, Third Military Medical University, Chongqing, People's Republic of China; 4 Department of Radiology, Southwest Hospital, Third Military Medical University, Chongqing, People's Republic of China; 5 Key Laboratory for NeuroInformation of Ministry of Education, School of Life Science and Technology, University of Electronic Science and Technology of China, Chengdu, People's Republic of China; Cuban Neuroscience Center, Cuba

## Abstract

Although there is increasing evidence suggesting that there may be subtle abnormalities in idiopathic generalized epilepsy (IGE) patients using modern neuroimaging techniques, most of these previous studies focused on the brain grey matter, leaving the underlying white matter abnormalities in IGE largely unknown, which baffles the treatment as well as the understanding of IGE. In this work, we adopted multiple methods from different levels based on diffusion tensor imaging (DTI) to analyze the white matter abnormalities in 14 young male IGE patients with generalized tonic-clonic seizures (GTCS) only, comparing with 29 age-matched male healthy controls. First, we performed a voxel-based analysis (VBA) of the fractional anisotropy (FA) images derived from DTI. Second, we used a tract-based spatial statistics (TBSS) method to explore the alterations within the white matter skeleton of the patients. Third, we adopted region-of-interest (ROI) analyses based on the findings of VBA and TBSS to further confirm abnormal brain regions in the patients. At last, considering the convergent evidences we found by VBA, TBSS and ROI analyses, a subsequent probabilistic fiber tractography study was performed to investigate the abnormal white matter connectivity in the patients. Significantly decreased FA values were consistently observed in the cerebellum of patients, providing fresh evidence and new clues for the important role of cerebellum in IGE with GTCS.

## Introduction

Genetically determined, idiopathic generalized epilepsy (IGE) constitutes nearly one third of all epilepsies and is typically characterized by absence, myoclonic and generalized tonic-clonic seizures (GTCS), alone or in varying combinations and severity, which may cause severe injuries even death [1], [2], [3], [4]. Although the diagnosis and confirmation of IGE is usually not difficult by combining electroencephalogram (EEG) monitoring and clinical information, locating the underlying abnormalities in the patient's brain is an extremely challenging task since neuroimaging abnormality can hardly be found by routine magnetic resonance imaging (MRI) examination [5], which baffles the treatment as well as the understanding of IGE. With the progress in structural and functional neuroimaging over the last decade, more and more studies suggest that there may be subtle abnormalities in IGE (reviewed in [6]), however, most of these previous imaging studies focused on the brain grey matter, leaving the underlying white matter abnormalities in IGE largely unknown.

Scientists generally accept that white matter plays a critical role in physiological mechanisms of brain function and neural signaling [7], [8]. Diffusion tensor imaging (DTI), which can explore the structure of white matter *in vivo*, can provide information about white matter integrity and pathology [9], [10], [11], [12], [13]. Fractional anisotropy (FA), which is derived from DTI, has been shown to reflect functionally relevant micro-structural properties of white matter, including axonal architecture, the extent of myelination and the density of axonal fibers comprising axonal bundles [14] and has therefore been interpreted as a measure of micro-structural integrity [15], [16]. Additionally, the continuity of individual white matter fibers in three-dimensional space can be estimated using white matter tractography [17], [18], [19], [20], [21], making tract-based analysis of white matter connectivity possible.

In this current work, we adopted multiple methods from different levels based on DTI to analyze the white matter abnormalities in IGE patients. First, we performed a voxel-based analysis (VBA) of the FA images to explore any abnormalities throughout the brain of IGE patients. Second, we used a tract-based spatial statistics (TBSS) method to explore the alterations within the white matter skeleton of the patients, which can overcome some drawbacks of VBA such as alignment issue and smoothing issue [22]. Third, we adopted region-of-interest [23] analyses based on the findings of VBA and TBSS to further confirm brain regions with abnormal structures in IGE. At last, considering the convergent evidences of the white matter abnormalities we found by VBA, TBSS and ROI analyses, a subsequent probabilistic fiber tractography study was employed to further investigate the abnormal white matter connectivity in the patients.

## Materials and Methods

### Subjects

Fourteen male patients with IGE (mean age  = 26 years; range  = 19∼36 years) and twenty-nine male age-matched healthy controls (mean age  = 27 years; range  = 21∼36 years) were included in this study. All the patients were consecutively recruited from the epilepsy clinic in Chongqing Southwest Hospital who showed no abnormity in routine MRI examines but had generalized spike-wave (GSW) or polyspike discharges with normal background in video-EEG monitoring. For each patient, clinical information including The National Hospital Seizure Severity Scale (NHS3) [24] was collected through interview with himself and his relatives who witnessed his epileptic seizure. Considering their seizure history and semiology as well as the results from video-EEG recording, all the patients were diagnosed by experienced clinicians to have IGE with GTCS only according to The International League Against Epilepsy (ILAE) classification [3]. The healthy controls were recruited by advertisement. All the subjects were right handed and Han Chinese in origin with no history of drug intoxication, encephalopathy, or traumatic brain injury. The demographic data of all patients can be found in Table 1.

**Table 1:** Demographic data of IGE patients.

Patient	Gender	Age (years)	Age of Onset (years)	Duration (years)	Type	Family history	EEG	AED	NHS3
1	M	34	22	12	GTCS	No	GSW	VPA	10
2	M	36	16	20	GTCS	No	GSW	PH	13
3	M	35	19	16	GTCS	No	GSW	VPA	12
4	M	28	15	13	GTCS	No	GSW	VPA	15
5	M	27	17	10	GTCS	No	GSW	CBZ/PH	12
6	M	19	15	4	GTCS	No	GSW	VPA/CBZ	10
7	M	25	23	2	GTCS	No	GSW	VPA	13
8	M	32	22	10	GTCS	No	GSW	CBZ	15
9	M	21	17	4	GTCS	No	GSW	CBZ	17
10	M	20	6	14	GTCS	No	GSW	PH	12
11	M	19	10	9	GTCS	No	GSW	CBZ	14
12	M	24	20	4	GTCS	No	GSW	PB	10
13	M	20	15	5	GTCS	No	GSW	CBZ/PH	14
14	M	25	9	16	GTCS	No	GSW	CBZ/VPA	10

Abbreviations: AED, antiepileptic drugs; VPA, sodium valproate; PH, phenytoin; CBZ, carbamazepine; PB, phenobarbital; M, male.

### Ethics Statement

After a full explanation, all subjects gave voluntary and informed consent in written form according to the standards set by the Ethical Committee of Third Military Medical University, who specifically approved this study.

### MRI data acquisition and preprocessing

DTI data of all the subjects were obtained on a SIEMENS Trio 3.0 Tesla scanner. A single shot echo planar imaging sequence (TR  = 6100 ms, TE  = 93 ms) was employed. The diffusion sensitizing gradients were applied along 64 non-collinear directions (b = 1000 s/mm^2^), together with a non-diffusion-weighted acquisition (b = 0 s/mm^2^). From each subject, 45 axial slices were collected. The field of view was 256 mm×256 mm; the acquisition matrix was 128×128 and zero filled into 256×256; the number of average was 1; and the slice thickness was 3 mm with no gap, which resulted in a voxel-dimension of 1 mm×1 mm ×3 mm. A 3D T1-weighted image for each subject was obtained using a magnetization prepared rapid gradient echo sequence (TR  = 2530 ms, TE  = 2.34 ms). The imaging parameters were a field of view of 256 mm ×256 mm, flip angle of 7°, and a voxel-dimension of 1 mm ×1 mm ×1 mm. In addition, T2-weighted axial/oblique coronal images and FLAIR oblique coronal images were acquired as part of the epilepsy examination. All images were submitted for visual analysis by experienced radiologists and received normal reports.

Both the DTI and T1-weighted data were visually inspected by two radiologists for apparent artifacts arising from subject motion and instrument malfunction. Distortions in the DTI caused by eddy currents and simple head motions were then corrected by FSL4.1 software (http://www.fmrib.ox.ac.uk/fsl). After correction, three-dimensional maps of the diffusion tensor and the FA were calculated using our in-house software (DTI Tracking System 1.0; http://www.ccm.org.cn). T1-weighted images of each subject were co-registered to the subject's non-diffusion-weighted image (b = 0 s/mm^2^) using the SPM8 package (http://www.fil.ion.ucl.ac.uk/spm/software/spm8), resulting in a co-registered T1 image (rT1) in DTI space.

### VBA of FA images

Utilizing SPM8 package, each subject's rT1 image (co-registered to DTI space, voxel-dimension of 1 mm×1 mm×3 mm) was first normalized to the T1 template in Montreal Neurological Institute (MNI) space. The normalization consisted of a 12 iteration linear transformation and a non-linear transformation with 7×8×7 basis functions. Parameters from this transformation were then applied to each subject's FA image, while re-sampling the volume into a voxel size of 2 mm ×2 mm ×2 mm. Further, each normalized FA image was spatially smoothed by an 8-mm full-width at half maximum Gaussian kernel to reduce the effect of misregistration in spatial normalization [25], [26].

We performed a two-sample *t-test* on the normalized FA images between the two groups in a voxel-based manner, using the statistical parametric mapping tool within SPM8. Also, correlations between the FA values and the clinical measurements (years of illness duration and NHS3 scores) were performed across the IGE patients using SPM8. The threshold value was set at *P*<0.0001 (uncorrected) with cluster size >30 voxels (2 mm ×2 mm ×2 mm ×30 = 240 mm^3^) for significance.

### TBSS analyses of FA images

TBSS analyses of the FA images was carried out using FSL4.1 software in the following steps [22]: first we aligned the FA images of all subjects to a pre-identified target image FMRIB58_FA (http://www.fmrib.ox.ac.uk/fsl/data/FMRIB58_FA) in standard MNI space by nonlinear registrations; next we transformed all the aligned FA images into 1 mm ×1 mm ×1 mm by affine registrations; then we created the mean FA image and its skeleton from all subjects including both the IGE patients and the healthy controls in MNI space, and each subject's FA was projected onto the skeleton; finally, voxel-wise statistic analyses were performed across subjects for each point on the common skeleton.

A two-sample *t-test* on the skeleton between the FA images of the IGE patients and the healthy controls was performed in a voxel-based manner using the Threshold-Free Cluster Enhancement (TFCE) method, which can enhance cluster-like structures without having to define an initial cluster-forming threshold or carry out a large amount of data smoothing [27]. The significance was determined with *P*<0.05 (FDR corrected). In order to maintain comparable with the VBA results as well as to minimize false-positive findings, we further threshold the TBSS results with cluster size >240 voxels (1 mm ×1 mm ×1 mm ×240 = 240 mm^3^, which is the same as we used in VBA study). In addition, correlation analyses were performed between the FA images of all IGE patients and their NHS3 scores as well as the illness duration using the same TFCE method and statistic threshold.

### ROI analyses of FA images

To better clarify our findings, we further selected ROIs considering the results of VBA and TBSS analyses and calculated the average FA value of each selected ROI subsequently for statistical analyses.

On one hand, we selected the involved brain regions found by VBA and TBSS in the DTI native space of each subject by utilizing the automated anatomical labeling (AAL) template [23] available with the MRIcro software (http://www.cabiatl.com/mricro/mricro), including the AAL regions of Cerebelum_6_R, Cerebelum_4_5_R, Cerebelum_Crus1_L and ParaHippocampal_L (The name of AAL regions are provided by the MRIcro software; “L” means that the brain region is located at the left hemisphere; “R” means right hemisphere.). In detail, each individual co-registered T1 image (rT1 in DTI space) had already been normalized to the T1 template in MNI space in our previous VBA study (see the VBA section). The resulting inverse transformation was then used to warp the AAL template from MNI space to the DTI native space in which the discrete labeling values were preserved by using a nearest neighbor interpolation method [28]. The inverse transformation was implemented using the SPM8 package.

On the other hand, we selected the brainstem region in the DTI native space of each individual by employing the FMRIB's Integrated Registration and Segmentation Tool (FIRST; http://www.fmrib.ox.ac.uk/fsl/first) available with the FSL4.1 software. First, the original T1 image of each subject (voxel size 1 mm ×1 mm ×1 mm) was fed into FIRST with the brainstem specified as output. Then, the transformation we used before resulting in a co-registered T1 image (from T1 to rT1) in DTI space was employed here again to warp the brainstem ROI obtained from FIRST into the DTI native space (voxel size 1 mm ×1 mm ×3 mm).

We notice that, instead of deriving ROIs directly from the clusters with significantly decreased FA values found by VBA or TBSS studies, we utilized the AAL template as well as brainstem segmentation method for our ROI selection and calculated the average FA value of the entire selected region, which may lead to false negative findings. Our argumentation for such ROI selecting scheme is that the clusters found by VBA and TBSS are not comparable with each other due to different registration method, different voxel dimensions as well as different statistical threshold, but involved the same AAL or brainstem regions. Our current scheme may help to reduce any bias resulted from using the clusters found by VBA or TBSS directly, while keeping the information they provided in consideration. All the ROIs mentioned above were showed in two randomly selected subjects (one IGE patient and one healthy control) to provide support for the validation of our ROI selection (Fig. S1).

Two-sample *t-tests* on the average FA values of the selected ROIs were performed between the IGE patients and the healthy controls. Also, correlations between the average FA values and the clinical measurements including years of illness duration and NHS3 scores were performed across the IGE patients.

### Probabilistic fiber tractography analyses

Considering the results of VBA and TBSS studies, which were performed in a whole brain manner, as well as the ROI study, which focused on several specific brain regions subsequently, the AAL regions of Cerebelum_6_R and Cerebelum_4_5_R were consistently observed with significantly decreased FA values in the IGE patients. However, whether the connectivity between these two regions and other AAL areas of the IGE patients are different from the healthy controls remains unclear.

We performed probabilistic fiber tractography analyses on the diffusion tensor images of all subjects using Cerebelum_6_R and Cerebelum_4_5_R as seed regions. In detail, we estimated the local probability distribution of fiber direction at each voxel using the Bayesian framework proposed by Behrens et al. [29], and a computation model capable of automatically estimating two fiber population within each voxel was employed [30]. Probabilistic tractography was applied by sampling 5000 streamlines per voxel. For each sampled fiber line, we drew a sample direction from the local distribution of fiber direction and then proceeded a fixed distance of 0.5 mm along this direction to a new position from which another sample direction was drawn. This propagation procedure will stop if the brain surface was reached or the fiber path looped back on itself. Therefore, the connectivity from a seed voxel *i* to another target voxel *j* was defined as the number of fibers passing through voxel *j* divided by the total number of fibers sampled from voxel *i* [30]. The idea of connectivity between voxels can be easily extended to the regional level. For a seed region with *n* voxels, 5000*n fibers were sampled (5000 fibers for each voxel). The number of fibers passing through a given region divided by 5000*n is defined as the connectivity from the seed region to this target region [30]. It should be noted that the connectivity from *i* to *j* is not necessarily equivalent to the one from *j* to *i* because that the tractography results are dependent on different seeding location. However, it has been demonstrated by previous study that these two connectivity values are highly correlated with each other [31]. So, only the connectivity from the seed region *i* to the target region *j* is used for our following statistical analyses. The connectivity we mentioned here, which was derived from probabilistic tractography based on DTI, has been well applied and supported by previous human brain studies [31], [32], [33], [34]. The above mentioned estimation of fiber direction distribution, subsequent probabilistic tractography and the calculation of regional connectivity were implemented using FSL4.1 software and our in-house scripts developed in the Matlab 7.8 platform.

The brain of each subject was segmented into 116 AAL regions using the same method we had employed in our ROI study for selecting AAL areas in the DTI native space (see the previous section of ROI analysis). AAL areas of Cerebelum_6_R and Cerebelum_4_5_R were selected as seed regions separately, and their connectivity to all the other 115 AAL regions was calculated. Subsequently, two-sample *t-tests* of the connectivity values were performed between the IGE patients and the healthy controls. Analyses of the correlation between the connectivity values and the clinical measurements were performed across the IGE patients.

## Results

### VBA study

**Figure 1:**
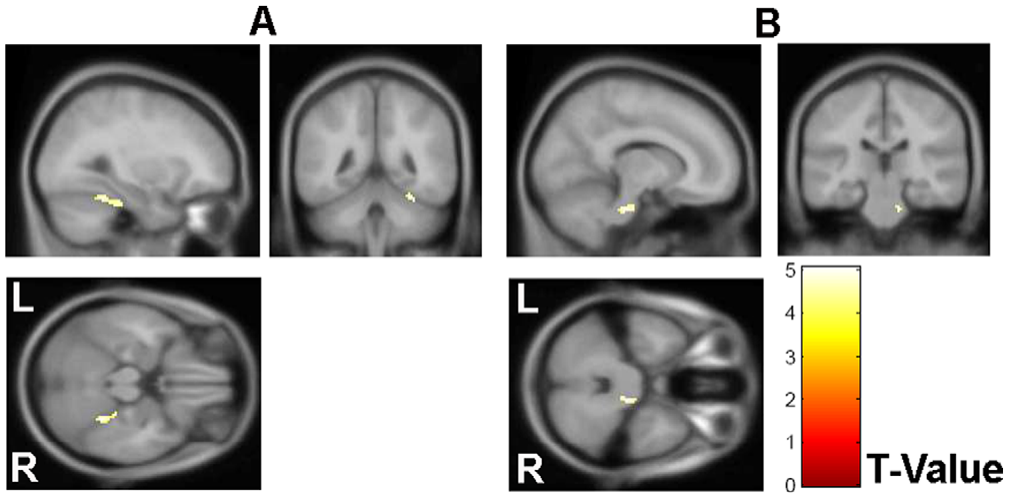
VBA result. Clusters with significantly decreased FA were overlaid on the MNI152 image for presentation. The t value of the stats images ranged from 0 (Red) to 5 (Yellow).

The FA images were compared in a voxel-wise manner by a two-sample *t-test* between the two groups. A significant decrease in the FA values was found to be located in the right cerebellum and the right brainstem in the IGE patients (Fig. 1). Specifically, significant alterations were found in areas involving the AAL regions of Cerebelum_6_R and Cerebelum_4_5_R (Fig. 1A) as well as the pons region located in the top portion of the right brainstem (Fig. 1B). No cluster with a significantly higher FA value was found in the patients group compared to the healthy controls. The MNI locations of the regions with reduced FA values in the IGE patients found by VBA are presented in Table 2. No significant correlation was found between the FA values and the clinical measurements, including the years of duration and the NHS3 scores, by VBA study.

**Table 2:** MNI locations of the regions with reduced FA found by VBA.

Corresponding anatomical locations	Cluster Size	Z score	Centroid voxel		
			x	y	z
Right Cerebellum (Cerebellum Anterior Lobe)	99	4.45	30	−46	−22
Right Brainstem (Pons)	43	4.37	12	−26	−32

*P*<0.0001 (uncorrected), cluster size >30 voxels.

Abbreviations: MNI, Montreal Neurological Institute; FA, Fractional Anisotropy.

### TBSS study

**Figure 2:**
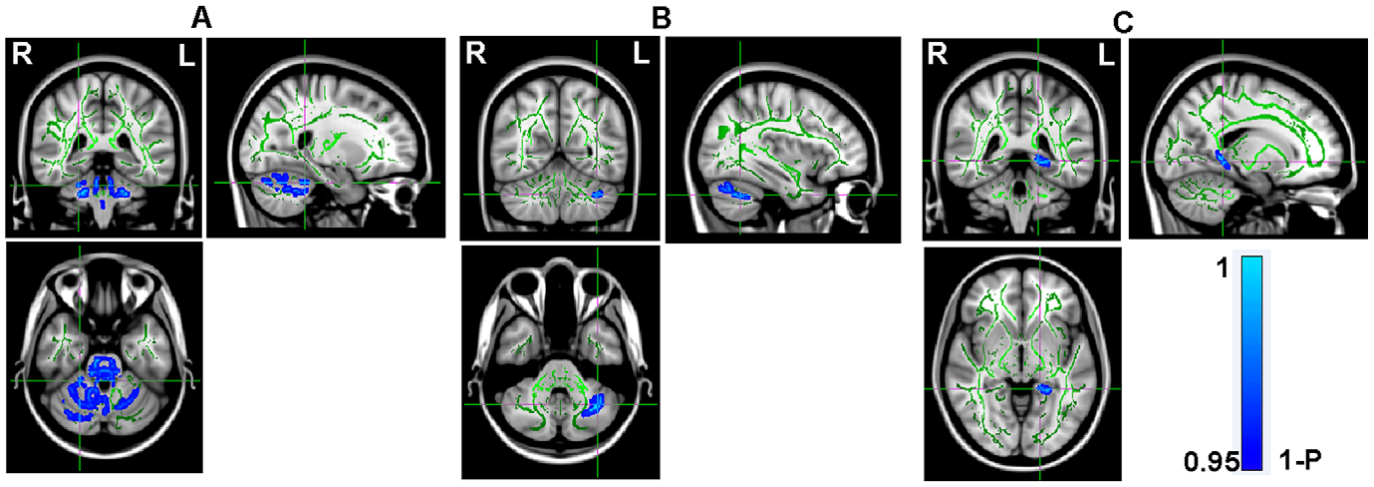
TBSS result. Mean FA skeleton across all subjects (Green) was overlaid on the MNI152 image for presentation; and the *P*_value image (it is actually 1-*P* for convenience of display) was overlaid on the skeleton; the range is from 0.95 (Blue) to 1 (Light Blue). Please note that, in order to visualize the skeletonised results of TBSS analysis easier, we thickened the results by filling it out into the local white matter fiber tracts.

A mean FA skeleton was created across all subjects (Green in Fig. 2), and significantly reduced FA values were detected in three clusters of the IGE patients located in right cerebellum, left cerebellum and left parahippocampal gyrus (Blue in Fig. 2 A, B and C). Specifically, significant alterations were found in areas involving the AAL regions of Cerebelum_6_R, Cerebelum_4_5_R (Fig. 2A) and Cerebelum_Crus1_L (Fig. 2B) as well as ParaHippocampal_L (Fig. 2C). No cluster with a significantly higher FA value was found in the patients group compared to the healthy controls. The MNI locations of the regions with reduced FA values in the IGE patients found by TBSS are presented in Table 3. Please note that, in Fig. 2, in order to visualize the skeleton results of TBSS analysis easier, we thickened the stats image by filling it out into the local white matter fiber tracts using the corresponding function of FSL4.1 software with the mean FA image generated across all subjects (the tbss_fill script; http://www.fmrib.ox.ac.uk/fsl/tbss). No significant correlation was found between the FA values and the clinical measurements by TBSS study.

**Table 3:** MNI locations of the regions with reduced FA found by TBSS.

Corresponding anatomical locations	Cluster Size	t value	Centroid voxel		
			x	y	z
Right Cerebellum (Cerebellum Anterior Lobe)	5541	1.54	24	−39	−28
Left Cerebellum (Cerebellum Anterior Lobe)	497	1.29	−35	−57	−36
Left Parahippocampa Gyrus (Limbic Lobe)	402	1.9	−17	−41	−4

*P*<0.05 (FDR corrected), cluster size >240 voxels.

Abbreviations: MNI, Montreal Neurological Institute; FA, Fractional Anisotropy; TBSS, Tract-based spatial statistics.

### ROI study

As shown in Table 4, the IGE patients showed significant lower FA values in AAL regions of Cerebelum_6_R and Cerebelum_4_5_R (*P*<0.05, FDR corrected). No significant result was found in AAL regions of Cerebelum_Crus1_L and ParaHippocampal_L as well as in the brainstem ROI. Also, no significant correlation was found between the average FA values of all selected ROIs and the clinical measurements we investigated including the NHS3 scores as well as the years of illness duration.

**Table 4:** Average FA value of five ROIs in 14 IGE patients and 29 healthy controls.

ROIs	FA, group mean (SD)		*P-* value (*Two samples t-test*) (Equal variances assumed)
	IGE (n = 14)	Controls (n = 29)	IGE *v.s.* Controls
**Cerebelum_6_R**	**0.156 (0.017)**	**0.169 (0.010)**	**0.002**
**Cerebelum_4_5_R**	**0.136 (0.016)**	**0.151 (0.012)**	**0.002**
Cerebelum_Crus1_L	0.150 (0.017)	0.159 (0.017)	0.117
ParaHippocampal_L	0.179 (0.027)	0.191 (0.019)	0.120
Brainstem	0.360 (0.022)	0.359 (0.083)	0.978

The threshold value was set at *P*<0.05 (FDR corrected) with equal variances assumed.

### Probabilistic tractography study

Figure S2 showed the results of brain segmentation in the DTI native space according to the AAL template as well as the probabilistic tractography of AAL regions Cerebelum_6_R and Cerebelum_4_5_R on two randomly selected subjects (one IGE patient and one healthy control). No significant difference of the connectivity values initiated from Cerebelum_6_R or Cerebelum_4_5_R to all the other 115 AAL regions was found between the patients and the healthy controls (*P*<0.05, FDR corrected).

**Figure 3:**
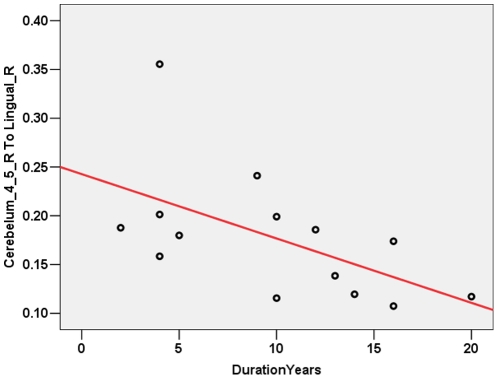
Tendency of correlation between connectivity and clinical measurements. The connectivity between AAL regions Cerebelum_4_5_R and Lingual_R was found to be negatively correlated to the years of illness duration across patients.

As shown in Fig. 3, among all the correlation analyses we performed, only the connectivity between AAL regions Cerebelum_4_5_R and Lingual_R was found to be negatively correlated to the years of illness duration across IGE patients when threshold at *P*<0.05 (uncorrected). In detail, the Pearson correlation coefficient was -0.564 and the *P*- value was 0.036.

## Discussion

In this study, comprehensive analyses based on DTI were performed in different levels to explore the white matter abnormalities in IGE patients with GTCS only. Significantly decreased FA values were consistently observed in the cerebellum of patients. Specifically, abnormalities were located in the right cerebella involving the AAL regions of Cerebellum_4_5_R and Cerebellum_6_R. Our findings provide fresh evidences supporting the important role of cerebellum in the pathology of IGE.

The cerebellum is a region of the brain that plays an important role in motor control. Specifically, the cerebellum does not initiate movement, but it contributes greatly to coordination, precision and accurate timing. There are neural pathways linking the cerebellum with the motor cortex and other movement-generating brain areas. Meanwhile, the cerebellum receives input from many sensory systems and many other parts of the brain as well as spinal cord; and integrates these inputs to fine-tune motor activity [35]. Because of its “fine-tuning” function, damage to the cerebellum does not cause paralysis, but instead produces disorders in fine movement, equilibrium, posture and motor learning [35]. Traditionally, epileptic seizures have been thought of as cerebrocortical phenomena, but there have been reports of seizures that were thought to originate within cerebellar gangliogliomas [36], [37], which suggests that at least in cases of cerebellar pathology, seizure activity may indeed commence within the cerebellum (reviewed in [38]). In one previous study of Gotman et al. performed on 15 IGE patients [39], a clear activation was seen in the cerebellum in response to epileptic discharges by recording the EEG during the fMRI examination. Also, intense involvement of the cerebellum during spike-and-wave discharges has been observed in an experimental model, independently of movement, with the suggestion that cerebellar neurons may contribute to spike-and-wave rhythmicity [40]. Although the DTI method we employed in this current study is not capable to confirm whether the abnormal structural we observed in cerebellum is the origin of the IGE patients' seizures, our findings suggest significant damage to the white matter integrity in the right cerebella of the patients, which indicates impaired fiber connectivity within the cerebellum as well as with other brain regions and might be closely related to their generalized tonic-clonic seizures symptom.

We notice that no cortical abnormalities were observed in the IGE patients of our current study which we believe is reasonable since we focused our investigation on the brain white matter using DTI methods. In fact, although not able to survive from the correction of multiple comparison, a tendency of negative correlation was found between the years of illness duration and the white matter connectivity initiated from cerebellum to the cortex by probabilistic fiber tracking method across all IGE patients, suggesting more impaired structural connectivity which may be related to longer illness duration. As reviewed by Krauss and Koubeissi [41], in addition to its potential epileptogenic effects, the cerebellum has also been proposed to have a role in seizure therapy because of its GABA-ergic Purkinje cells output and relay input into motor cortex, and has been investigated as targets for brain stimulation in the treatment of epilepsy in a number of experimental animal and human studies. The Purkinje cell inhibitory output and widespread cortical projections supports the possible role of cerebellar stimulation to reduce epileptogenic acitivty [41]. After years of experience with animal work and uncontrolled clinical trials, Cooper first developed electrical stimulation for the treatment of generalized cerebellar and partial seizure disorders in humans, and reported a 50% or greater reduction of seizures in petients treated with chronic anterior cerebellar stimulation [42], [43]. Overall, nearly 70% of the patients who are reported with cerebellar stimulation in the published literature had reduction in seizures [41]. However, studies of the influence of cerebellar stimulation on animal models of epilepsy and human clinical trials are conflicting, suggesting a variable influence on seizures [41]. Although the results from previous studies of chronic cerebellar stimulation treatment for epilepsy are encouraging, a variety of clinical issues remain unsettled, including which seizure types might be best treated, which stimulation parameters are optimal and what the physiological rationales for the treatment are. Our findings of cerebellum structural abnormalities in IGE patients with GTCS only suggest that cerebellum stimulation might be helpful for this specific kind of subjects. However, we should be cautious when explaining our findings and further studies on animal model as well as human clinical trials will be extremely necessary to confirm our speculations.

We acknowledge that there are limitations and methodological issues in our current study. First, some of our findings were uncorrected for multiple comparisons including the results of VBA and probabilistic fiber tracking analyses. More significant results may be achieved if more subjects can be included in future studies. Also, the relatively small sample size makes it inadequate to control for variables such as medications, seizure burden and frequency of discharges across patients. Second, the value of connectivity between two brain regions we calculated by probabilistic fiber tracking method does not necessarily indicate that there is an actual fiber which connects the two regions directly. Rather, it suggests the possibility of the existence of certain pathway between these two regions, which may further indicate the ability of signal transfer between them. Since we only performed probabilistic fiber tracking on two cerebellar AAL regions based on our previous findings from VBA, TBSS and ROI analyses, we can not rule out the possibility that there might be other abnormal connectivity in the IGE patients. Especially, our current investigation is not able to characterize the interaction and organization among all brain regions in the IGE patients. Future studies from the whole brain network point of view will be necessary for better understanding the mechanism of IGE. Finally, although all the patients included in this study are relatively pure considering their seizure type, age and gender, there may still be genetic differences among them. The combination of neuroimaging and genetic studies in the future will extremely help for better understanding the pathology of IGE.

In conclusion, convergent results were observed by multiple analyses based on DTI in our current study, showing that there were structural abnormalities in the cerebellum of the IGE patients with GTCS only. Our findings suggest that there was damage to the white matter integrity of the patients' cerebellum which may be related to their seizure symptom. Our study provides fresh evidences for the important role of cerebellum in IGE with GTCS and may give new clues for future studies.

## Supporting Information

Figure S1 **Five ROIs in the native DTI space of two randomly selected subjects.** Selected ROIs (Yellow) were overlaid on the rT1 image of each subject for presentation; (A) Cerebelum_6_R; (B) Cerebelum_4_5_R; (C) Cerebelum_Crus1_L; (D) ParaHippocampal_L; (E) Brainstem.
(TIF)

Figure S2 **Probabilistic tractography results on two randomly selected subjects.** (A) Transformed AAL template overlaid on the rT1 image of the randomly selected individual in the DTI native space. The homologous brain regions in AAL template were coded in different colors because the areas in the left and right hemispheres were considered separately. (B) Connectivity between Cerebelum_6_R and other AAL regions resulted from probabilistic tractography. (C) Connectivity between Cerebelum_4_5_R and other AAL regions resulted from probabilistic tractography. The color represents the resulting connectivity value (Yellow > Red).
(TIF)
