# Supplementary figures and images for: Cerebellum Abnormalities in Idiopathic Generalized Epilepsy with Generalized Tonic-Clonic Seizures Revealed by Diffusion Tensor Imaging
Source: PLoS One. 2010 Dec 21;5(12):e15219. doi: 10.1371/journal.pone.0015219 (PMC3006341; doi:10.1371/journal.pone.0015219)

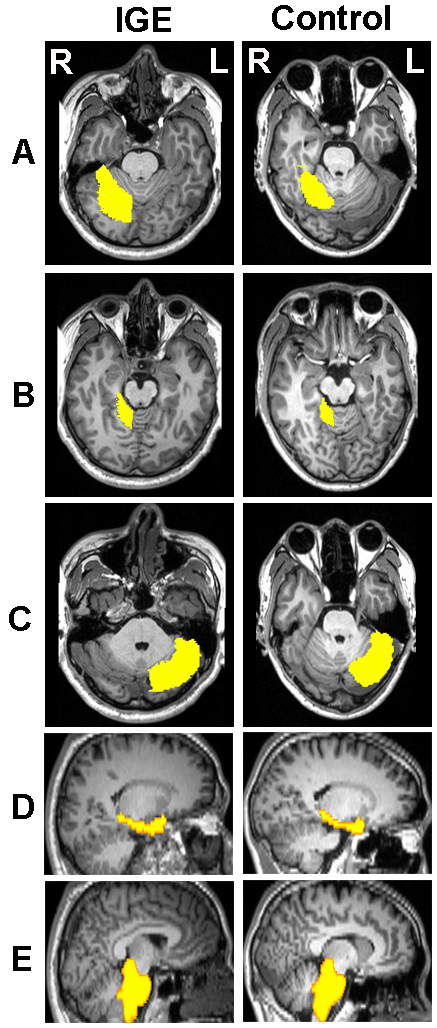

Supplement: Figure S1 — Five ROIs in the native DTI space of two randomly selected subjects. Selected ROIs (Yellow) were overlaid on the rT1 image of each subject for presentation; (A) Cerebelum_6_R; (B) Cerebelum_4_5_R; (C) Cerebelum_Crus1_L; (D) ParaHippocampal_L; (E) Brainstem. (TIF) [file pone.0015219.s001.tif]

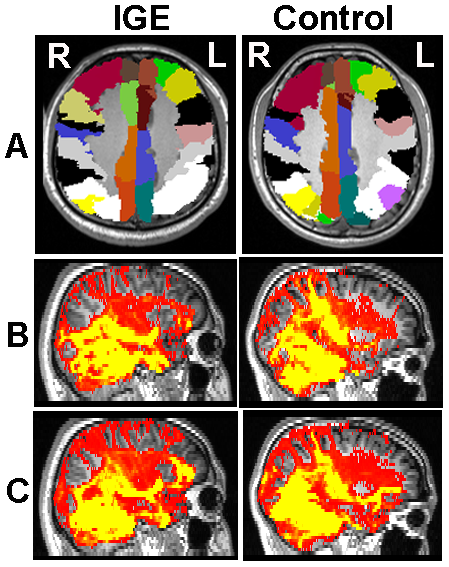

Supplement: Figure S2 — Probabilistic tractography results on two randomly selected subjects. (A) Transformed AAL template overlaid on the rT1 image of the randomly selected individual in the DTI native space. The homologous brain regions in AAL template were coded in different colors because the areas in the left and right hemispheres were considered separately. (B) Connectivity between Cerebelum_6_R and other AAL regions resulted from probabilistic tractography. (C) Connectivity between Cerebelum_4_5_R and other AAL regions resulted from probabilistic tractography. The color represents the resulting connectivity value (Yellow > Red). (TIF) [file pone.0015219.s002.tif]
